# Bioactivities of *Geranium wallichianum* Leaf Extracts Conjugated with Zinc Oxide Nanoparticles

**DOI:** 10.3390/biom10010038

**Published:** 2019-12-26

**Authors:** Banzeer Ahsan Abbasi, Javed Iqbal, Riaz Ahmad, Layiq Zia, Sobia Kanwal, Tariq Mahmood, Canran Wang, Jen-Tsung Chen

**Affiliations:** 1 Department of Plant Sciences, Quaid-i-Azam University, Islamabad 45320, Pakistan; benazirahsanabbasi786@gmail.com (B.A.A.); tmahmood.qau@gmail.com (T.M.); 2 College of Life Sciences, Shaanxi Normal University, Xi’an 710119, China; riaz17qau@gmail.com; 3 Superconductivity and Magnetism Laboratory, Department of Physics Quaid-i-Azam University, Islamabad 45320, Pakistan; layiqxia@gmail.com; 4 Department of Zoology, University of Gujrat, Sub-Campus Rawalpindi, Punjab 46300, Pakistan; sobiakanwal16@gmail.com; 5 Department of Biochemistry and Molecular Cell Biology, Shanghai Key Laboratory for Tumor Microenvironment and Inflammation, Shanghai Jiao Tong University School of Medicine, Shanghai 200025, China; canranw@gmail.com; 6 Department of Life Sciences, National University of Kaohsiung, Kaohsiung 811, Taiwan

**Keywords:** Zinc oxide nanoparticles, anticancer, antileishmanial, antimicrobial, biocompatibility

## Abstract

This study attempts to obtain and test the bioactivities of leaf extracts from a medicinal plant, *Geranium wallichianum* (GW), when conjugated with zinc oxide nanoparticles (ZnONPs). The integrity of leaf extract-conjugated ZnONPs (GW-ZnONPs) was confirmed using various techniques, including Ultraviolet–visible spectroscopy, X-Ray Diffraction, Fourier Transform Infrared Spectroscopy, energy-dispersive spectra (EDS), scanning electron microscopy, transmission electron microscopy, and Raman spectroscopy. The size of ZnONPs was approximately 18 nm, which was determined by TEM analysis. Additionally, the energy-dispersive spectra (EDS) revealed that NPs have zinc in its pure form. Bioactivities of GW-ZnONPs including antimicrobial potentials, cytotoxicity, antioxidative capacities, inhibition potentials against α-amylase, and protein kinases, as well as biocompatibility were intensively tested and confirmed. Altogether, the results revealed that GW-ZnONPs are non-toxic, biocompatible, and have considerable potential in biological applications.

## 1. Introduction

Nanotechnology is one of the important thriving and rapidly developing interdisciplinary sciences involving a combination of knowledge from materials science, physics, chemistry, and biology, etc. The term “nano” is a Greek word which means “extremely small or dwarf” in size range of about one to one-hundred nanometers. NPs possess unique and fascinating magnetic, electrical, optical, and chemical properties with small size, different shapes, and surface effects compared to bulk materials [1,2,3]. NPs possess breakthrough applications in different fields such as food, agriculture, medicine, cosmetics, energy, environment, and many more [4,5]. Among the different metal nanoparticles (MNPs), zinc oxide nanoparticles (ZnONPs) has received due attention due to their multifunctional and tunable nature. ZnONPs possess 3.37 eV direct band gap with high excitation energy (60 meV), which make it perfect to be utilized in UV photodetector, transistors, and semiconductor diodes [6,7,8]. In addition, ZnONPs are used in the field of bio-imaging, drug delivery, mineral based sunscreens, lotions, and ointments; biomedicine, especially in the fields of anticancer and antibacterial fields, which are involved with their potent ability to trigger excess reactive oxygen species (ROS) production, release zinc ions, and induce cell apoptosis [9].

Numerous physical and chemical routes have been developed to synthesize ZnONPs with disparate morphologies and sizes [10,11,12,13]. These physical synthesis routes face numerous problems; they need high energy, costly instruments, and require high temperature and pressure [14]. Meanwhile, chemical approach involves synthesis of ZnONPs using several reducing agents, costly metal salts, toxic solvents, and reductants. These physical and chemical methods are not only expensive at industrial level production, but also possess potential environmental and biological hazards [15,16,17,18]. In contrast, biological/biogenic synthesis of NPs is an emerging area and economically feasible option in the field of “green chemistry” [19,20,21,22]. It is considered to be simple, safer, greener, easily scaled up, non-toxic, eco-friendly, energy-efficient, cost-effective, and performed at room temperature and pressure in the absence of non-hazardous solvents and reductants [19,20,21]. Biological fabrication of NPs can be accomplished using different bacteria, algae, diatoms, and medicinal plants [22,23]. The major disadvantage associated with microbial sources is maintaining contamination free environment, high isolation cost and their maintenance in culture media. Therefore, plants (phytofabrication) promise to be an excellent route in the formation of NPs due to simplicity, replacing chemicals to lessen or even remove materials that are harmful to human health and the ecosystem [24]. The use of a plant-extract in the synthesis of NPs has recently gained significant popularity [20,25]. The phytochemicals available in plants act as a reducing agents, leading to a synthesis of capped NPs, and thus reducing costs and chemical reagents [25,26]. Further, phytochemicals and aqueous environment replace many harmful organic/inorganic solvents and chemical compounds [26]. Currently, plant extracts of various plant parts have been used in the biofabrication of NPs [25]. Plant extracts possess potential biological activities in biomedical applications due to the presence of different phytomolecules: alkaloid, flavonoid, phenolic, terpenes, amino acids, and vitamins may function as strong reducing and stabilizing agents, reducing cost and eliminating the use of toxic chemicals agents [27,28,29,30].

Numerous investigations have focused on the green synthesis of ZnONPs and have reported different biological activities [31,32,33,34]. Considering the importance of green synthesis, in the present study, ZnONPs have been synthesized using GW leaves extract. The plant possesses many therapeutic application in the treatment of rheumatism, leucorrhoeas, arthritis, gonorrhea, heart, and liver related problems. Among the various plants used in the formation of green NPs, GW was selected due to large amount of bioactive compounds; ursolic acid, herniarin, stigmasterol, β-sitosterol, herniarin, etc. which can help in the reduction, stabilization, and capping of metal ions [35]. 

The aim of the current research study was to biosynthesize ZnONPs (Zinc nitrate hexahydrate) using natural GW leaves extract without utilizing any surfactants and chemical solvents. The reaction condition and synthesis protocol have been discussed comprehensively. To the best of our knowledge, this is the first research study reporting green fabrication of ZnONPs using GW leaves extract. Furthermore, ZnONPs were extensively studied using various characterizations techniques, followed by investigations of its diverse bio-potentials using number of activities and assays.

## 2. Experimental

### 2.1. Plant Sampling and Extract Preparation

*Geranium wallichianum* (Geraniaceae) was collected in its flowering stage from Nathiagali Mountains (34°04′ N 73°23′ E), Khyber Pakhtunkhwa, Pakistan. The soil is typically moist, loamy, and very shallow. The weather remains cool, pleasant and foggy in summers, cold and chilly in winters with heavy snow [35]. The leaves of GW were thoroughly washed with deionized water, shade dried for 10–15 days so that water content gets removed entirely. The leaves were crushed to powder and preserved in dry and airtight container. The plant extract was prepared by mixing 10 g of GW fresh leaves powder with 250 mL distilled water. The mixture was heated for 2 h at 80 °C under continuous stirring. The resulting solution was cool down at room temperature and filtered three times utilizing Whatman filter papers. The resulting filtered extract was preserved at 4 °C for future use in the biofabrication of ZnONPs.

### 2.2. Synthesis of ZnONPs

**Figure 1:**
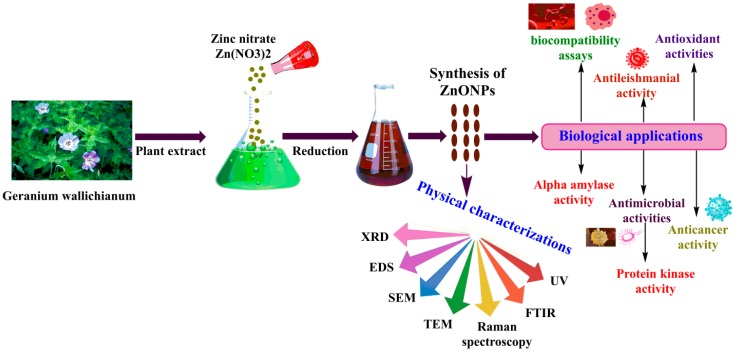
The process of green synthesis of leaf extract-conjugated zinc oxide nanoparticles.

Synthesis of ZnONPs was successfully performed by reducing Zn(NO_3_)_2_·6H_2_O using GW leaf extract. In detail, 50 mL filtered GW leaves extract was taken and mixed with 3 gm Zn(NO_3_)_2_·6H_2_O salt, heated at 60 °C, and continuously stirred at 500 rpm for 2 h. The obtained solution was centrifuged at 12,000 rpm/30 min. The pellet containing ZnONPs was carefully washed 3–4 times with double distilled water. The obtained powder assumed as ZnONPs was placed in an oven at ~100 °C for 3 h. Further, ZnONPs were calcined in a furnace to obtain crystalline ZnONPs. The calcined ZnONPs were kept in a cool, dry, and dark place, and their characterizations were performed. Figure 1 shows a study layout of ZnONPs from synthesis to characterization and biological applications.

### 2.3. Characterization of ZnONPs

The biogenic ZnONPs were characterized morphologically, physically, and chemically using various analytical techniques. The bio reduction of zinc ions to ZnONPs was determined by measuring the absorption spectra of reaction solutions via UV-4000 UV-Vis spectrophotometer (Germany) between 200–700 nm. The morphology of the ZnONPs was analyzed through SEM (EM (NOVA FEISEM-450 applied with EDX detectors). TEM analyses was performed to study size and shape of ZnONPs. The average particle size and particle size distribution of ZnONPs were studied via DLS system, using Malvern Zetasizer Nano (Malvern instrument). FTIR (Alpha, Bruker, Germany) analysis was performed between 500–4500 cm^−1^ to study diverse types of functional groups which are responsible in reduction and effective stabilizations for ZnONPs using various modes of vibrations. The crystalline structure of biogenic ZnONPs was determined by PANalytical XRD (Netherland) and crystal size was calculated. The Raman spectroscopy analyses was performed for ZnONPs to study their vibrational properties. The EDS analyses were done to detect the elemental composition of ZnONPs.

### 2.4. Bio-Potentials of Green GW-ZnONPs

#### 2.4.1. Metabolic Activity of GW-ZnONPs against HepG2 Cells

The MTT cytotoxicity assay was performed according to the previously described method to assess the survival (viability) of the HepG2 (liver cancer cells) by assessing the mitochondrial activity inside the cells after treatment with ZnONPs. For this purpose, HepG2 cells were cultured in DMEM media provided with 10% FBS, 1% Pen-Strep. Cells were seeded in 96-well plates and placed in 5% CO_2_ incubator at 37 °C for cell attachment. To assess the cytotoxicity potentials of green ZnONPs, HepG2 cells were treated with different doses of zinc oxide nanoparticles (7.8125–1000 µg/mL) for 48 h and MTT assay was performed. The ZnONPs treated cells were placed in DMEM medium and placed in an incubator for 24 h. After incubation, 100 µL MTT solutions was loaded and set aside for ~2 h. The MTT dye reacted with the oxidoreductase enzyme present in the mitochondria and was then converted into crystals of formazan. This process occurred only in viable cells. Further, the formazan crystals were dissolved with dimethyl sulfoxide (DMSO) and its absorbance was measured using a plate reader at 570 nm. Untreated cells were considered as control, while % inhibition of HepG2 cell lines treated with the synthesized ZnONPs nanoparticles was calculated using the formula below.

(1) $$\%\mathrm{inhibition}=\frac{1-\mathrm{OD}\;\mathrm{of}\;\mathrm{sample}}{\mathrm{OD}\;\mathrm{of}\;\mathrm{control}}\times 100$$

#### 2.4.2. Antileishmanial Potential of ZnONPs

The cytotoxicity potentials of ZnONPs were further evaluated using *Leishmania tropica* “KWH23 strain” (both amastigotes and promastigotes culture) utilizing cytotoxicity assay [30,36]. The MI99 media was used added with 10% FBS to culture leishmanial parasites. The leishmanial parasites were treated with various concentrations of ZnONPs (1–200 μg/mL) to evaluate their antileishmanial potentials. During the experiment, Amphotericin-B was considered positive and DMSO as negative control. The Leishmanial parasites in 96 well plates were treated with various concentrations of ZnONPs and kept in 5% CO_2_ incubator for 72 h at 24 °C, and absorbance was measured at 540 nm. After treatment, all living leishmanial parasites were counted under a microscope and IC_50_ values were recorded. Median lethal concentration (IC_50_) was calculated using GraphPad software, while percent inhibition was calculated using the following formula:

(2) $$\%\mathrm{inhibition}=\frac{1-\mathrm{sample}\;\mathrm{absorbance}}{\mathrm{absorbance}\;\mathrm{of}\;\mathrm{control}}\times 100$$

#### 2.4.3. Alpha Amylase (AA) Inhibition Potential

The AA inhibition potential of biogenic ZnONPs was determined using a previously described method [36]. The reaction mixture for the activity was made by mixing 25 μL of AA enzyme, 10 μL ZnONPs, 40 μL starch solutions, and 15 μL FBS. The reaction solution with all component was incubated at 50 °C for 30 min by adding 1 M HCL (20 µL) and iodine solutions (90 µL). The acarbose was utilized as positive and distilled water was utilized as negative control during experiment. Median lethal concentration (IC_50_) was calculated using GraphPad software while percent inhibition was calculated using the following formula:

(3) $$\%\mathrm{inhibition}=\frac{\mathrm{sample}\;\mathrm{absorbance}-\mathrm{absorbance}\;\mathrm{of}\;\mathrm{negative}\;\mathrm{control}}{\mathrm{absorbance}\;\mathrm{of}\;\mathrm{blank}-\mathrm{absorbance}\;\mathrm{of}\;\mathrm{negative}\;\mathrm{control}}\times 100$$

#### 2.4.4. Protein Kinase (PK) Inhibition Potential

The ZnONPs were also evaluated for its PK inhibition potential using *Streptomyces* 85E strain according to a previously published method [37]. The PK inhibition activity was conducted in a sterile environment. The SP4 minimal media was utilized to prepare even lawns of *Streptomyces*. 10 µL of ZnONPs loaded on filter discs were placed on the petri plates to determine their PK inhibition potential. The surfactin was taken as positive and DMSO as negative control. To target the growth of *Streptomyces* 85E strain, incubation was performed at 30 °C for 72 h. After 24 h, clear and bald zones appeared around the discs, which showed spores inhibition and mycelia development. Finally, zone of inhibition (ZOI) were measured.

#### 2.4.5. Antifungal Assays of ZnONPs

Fungicidal potentials of biogenic ZnONPs were investigated against different fungal strains via disc diffusion method. Before fungicidal assay was performed, fungal spores were sub-cultured in Sabouraud Dextrose liquid media and kept in an incubator for 24 h at 37 °C. The liquid cultures of different fungal strains were adjusted to OD of 0.5. Further, SDA solid media was made for culturing fungal strains and poured into petri plates. Filter discs laden with different concentrations of ZnONPs were kept on media plates. The Amphotericin-B was taken as positive and DMSO as negative controls. After loading test samples and both positive and negative controls, fungal plates were kept in an incubator for ~48 h at 28 °C to observe the ZOI. The fungal strains were treated with various concentrations of ZnONPs ranging from 31.25–1000 µg/mL, and their MIC value was recorded to determine their antifungal potential.

#### 2.4.6. Antibacterial Activity of ZnONPs

The bacterial inhibition potential of GW-ZnONPs were evaluated using various bacterial strains through discs-diffusion method. Before the activity was conducted, bacterial strains were sub-cultured overnight in nutrients broth media and incubated at 37 °C for 24 h. To determine the antibacterial potency of ZnONPs, an overnight culture of bacterial strains was spread on pre-prepared agar media and allowed to dry for 5 min. Subsequently, filter discs laden with different concentration of ZnONPs (31.25–1000 µg/mL) were dried and kept on surface of plates. The plates were kept in incubator at 37 °C for 24 h and observed for ZOI. The standard antibiotic oxytetracycline was taken as positive and 5% DMSO as negative control. Further, MIC values of GW-ZnONPs were determined by calculating ZOI.

#### 2.4.7. Antioxidant Capacities

The radical scavenging potential of ZnONPs was determined using spectrophotometric procedure. The working solution was prepared by mixing DPPH (2.4 mg) into 25 mL of methanol as free radicals. Before the activity was started, various concentrations (1–200 µg/mL) of ZnONPs were prepared and evaluated for their antioxidant potential. The ascorbic acid (AA) was taken as positive and DMSO as negative control. The 200 µL of reaction mixtures was comprised of 180 µL of reagent solution and 20 µL ZnONPs sample. The reaction mixture was then kept for 2 h under dark conditions and absorbance of reaction mixture was measured at 517 nm. The scavenging potential of GW-ZnONPs on free radicals are presented as follows:

(4) $$\%\;\mathrm{DPPH}\;\mathrm{scavenging}\;=\;1\;-\;(\frac{\mathrm{Absorbance}\;\mathrm{of}\;\mathrm{sample}}{\mathrm{Absorbance}\;\mathrm{of}\;\mathrm{control}})\;\times \;100$$

The antioxidant potential of ZnONPs was further studied by total antioxidant capacity (TAC) using previously described phosphomollybdenum method [38]. The absorbance was recorded at 695 nm and results were indicated as microgram equivalent of AA per/mg of test samples. AA was used as positive control and DMSO as negative control. Furthermore, total reducing power (TRP) of the asynthesized ZnONPs were studied using Potassium-ferricyanide procedure [39]. AA was taken as positive and DMSO was taken as negative controls. The absorbance of mixture solutions was recorded at 630 nm. The reducing power of asynthesized ZnONPs was measured as AA equivalents per milligrams (AAE/mg). 

#### 2.4.8. Biocompatibility of ZnONPs with Human Macrophages

The biocompatible nature of green ZnONPs were investigated using human macrophages via previously used method [40]. The macrophages were cultured in RPMI media supplemented with FBS (10%), Herpes (25 mM), antibiotics (Pen-Strep. Further, macrophages were seeded and cultured in 96-well plates, kept in 5% CO_2_ incubator for 24 h for cell attachment. The macrophages were exposed to various concentrations of biogenic ZnONPs (1–200 µg/mL) for 24 h. The absorbance was measured and % inhibition was calculated using the equation below.

(5) $$\%\;\mathrm{inhibition}\;=\;\frac{1\;-\;\mathrm{Absorbance}\;\mathrm{of}\;\mathrm{sample}}{\mathrm{Absorbance}\;\mathrm{of}\;\mathrm{control}}\;\times \;100$$

#### 2.4.9. Biocompatibility of ZnONPs with Human RBCs

The biocompatible nature of asynthesized ZnONPs was further confirmed using human RBCs through previously described method [41]. For hemolytic assay, 1 mL of fresh human RBCs was taken and stored in EDTA falcon to avoid blood coagulation. Further, centrifugation was performed for human RBCs at 12,000 rpm for 10 min. The erythrocytes suspension was made by adding 200 µL erythrocytes into 9.8 mL of PBS (pH 7.2). The 100 µL erythrocytes suspensions was treated with various doses of ZnONPs and incubated at 35 °C for 1 h. Further, centrifugation was performed at 12,000 rpm and supernatants was removed. Further, the supernatant was shifted into 96-well-plate and hemoglobin release was studied at 540 nm. During experiment, Triton X-100 was utilized as positive and DMSO as negative control. The results are calculated as % hemolysis produced by different concentration of ZnONPs and can be calculated employing the formula below:

(6) $$\%\;\mathrm{hemolysis}\;=\;\frac{\mathrm{Sample}\;\mathrm{abs}-\mathrm{Negative}\;\mathrm{control}\;\mathrm{abs}}{\mathrm{Positive}\;\mathrm{control}\;\mathrm{abs}-\mathrm{Negative}\;\mathrm{control}\;\mathrm{abs}}\;\times \;100$$

## 3. Results and Discussion

### 3.1. Biosynthesis of ZnONPs

**Figure 2:**
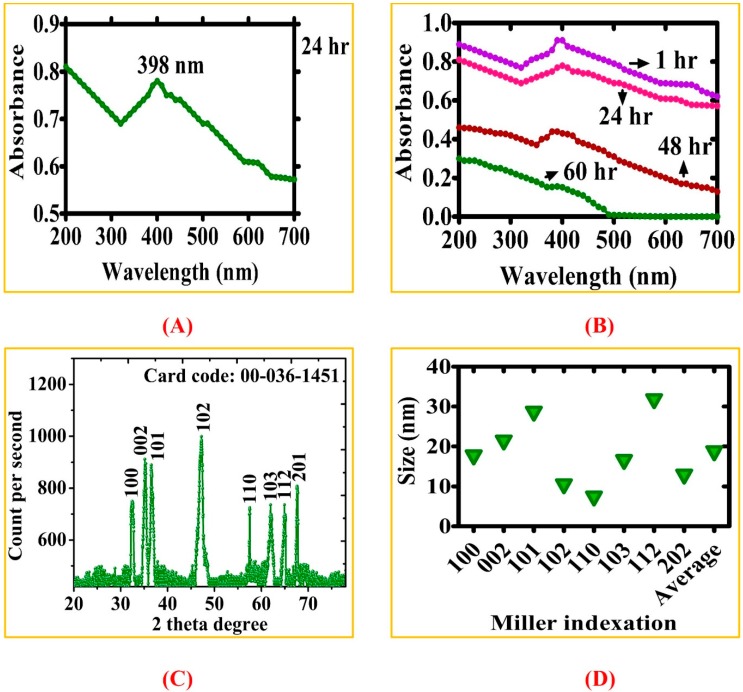
UV and XRD spectra analysis for biogenic ZnONPs (**A**) UV visible spectra (**B**) Stability of biosynthesized ZnONPs (**C**) XRD spectra of *Geranium wallichianum* mediated ZnONPs (**D**) Size calculation via Scherer approximation.

In this present study, ZnONPs were rapidly fabricated using GW leaves extract as a bioreductant and stabilizing agents. Different characterization techniques were performed to determine the formation of zinc oxide nanoparticles. The progress of synthesis of ZnONPs was detected by color change after the addition of precursor salt to plant extract at 60 °C. The color change in solutions (reddish black) signal the biosynthesis of ZnONPs. This color change in the solution is due to surface plasmon resonance (SPR) [42]. To confirm the stable nature of asynthesized ZnONPs, 1 mg/mL solutions of nanoparticles was prepared and sonicated for ~40 min. The turbid colloidal suspension was allowed to remain stable for 48 h, and SPR was observed with varied time interval. The wavelength scale was fixed between 200–700 nm and solution was scanned between this range. The UV spectra showed absorption peak at 398 nm, indicating that colloidal suspension remained stable for 48 h. The reduction in absorption peak was noticed after 60 h which showed settlement of NPs at the bottom. The UV-Vis spectrum for green ZnONPs are presented in Figure 2A,B. The XRD configurations of thermally annealed green GW-ZnONPs are presented in Figure 2C. The XRD analysis has confirmed the crystalline nature of ZnONPs. The resulting Bragg peaks were in accordance with single and pure phase hexagonal zincite with JCPD card no: 00–036–1451. The XRD spectra showed different distinct diffraction peak with 2Ø value of 32.44, 35.13, 37.34, 47.21, 57.51, 62.58, 65.76 and 67.61, which are corresponding to (100), (002), (101), (102), (110), (103), (112) and (201) Bragg’s reflections. XRD spectrum obtained indicates the absence of impurities. The presence of these peaks are due to leaves extract containing organic compounds, thus play significant role in reduction of zinc ions and stabilization of resultant ZnONPs [42]. The average size calculated as ~18 nm. The XRD configuration for ZnONPs are consistent with earlier studies using green synthesis protocol [43,44].

**Figure 3:**
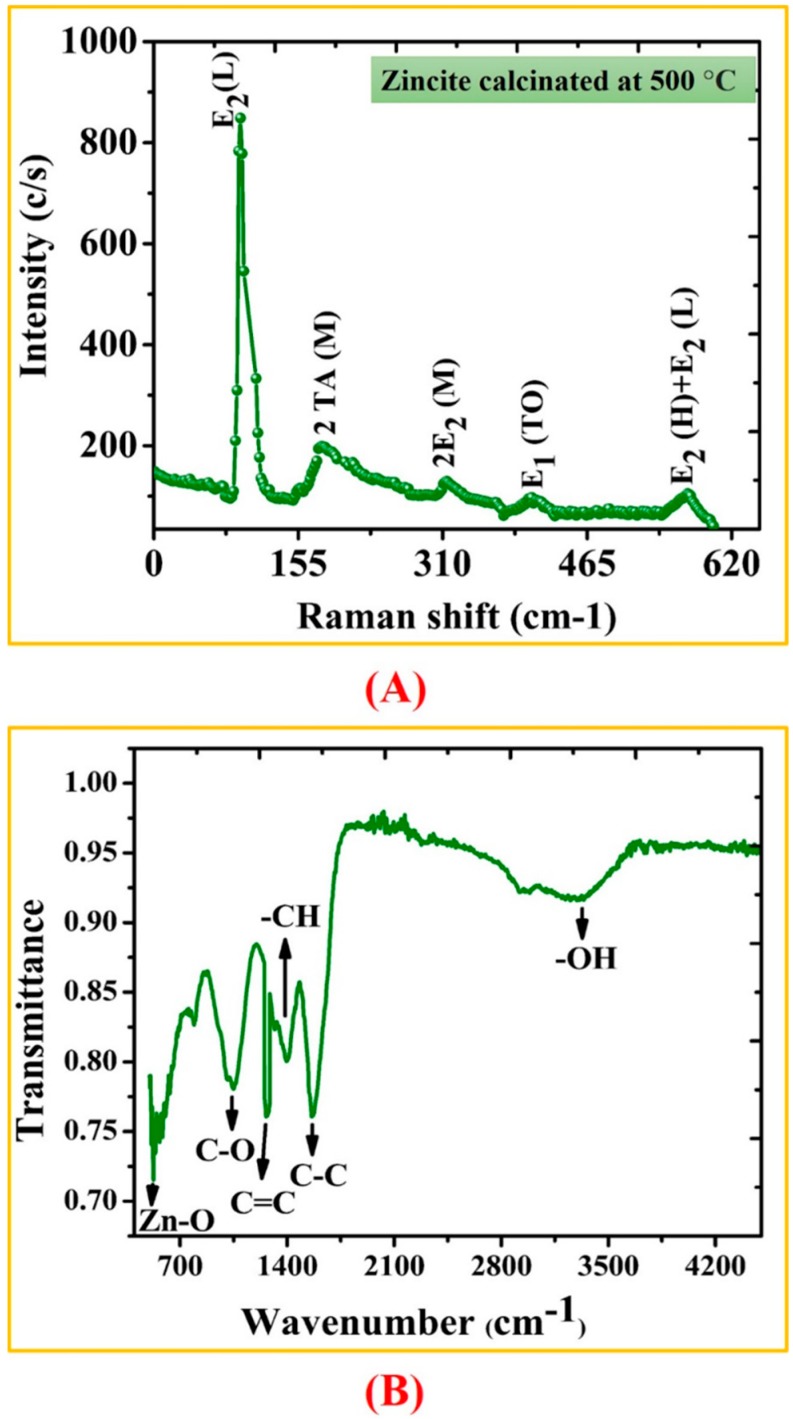
(**A**) Raman spectra of the ZnONPs biosynthesized using zinc nitrate hexahydrate as precursor (**B**) FTIR spectra of ZnONPs.

**Figure 4:**
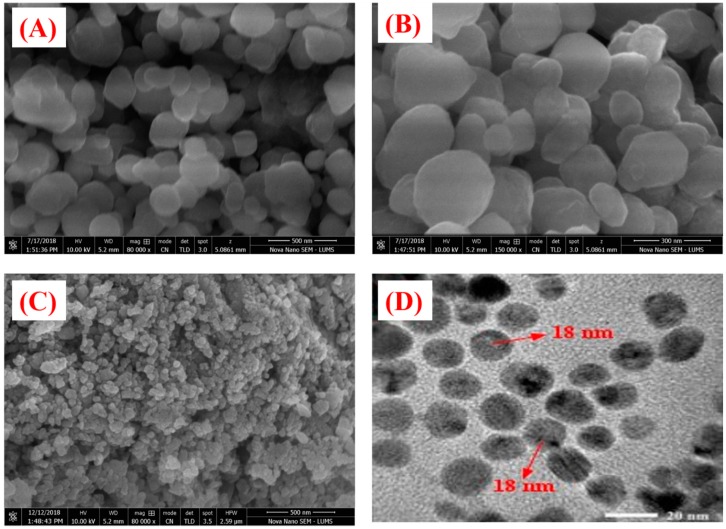
SEM and TEM images of *Geranium wallichianum* mediated ZnONPs using zinc nitrate as a precursor (**A**–**C**) HR-SEM images (**D**) TEM image.

**Figure 5:**
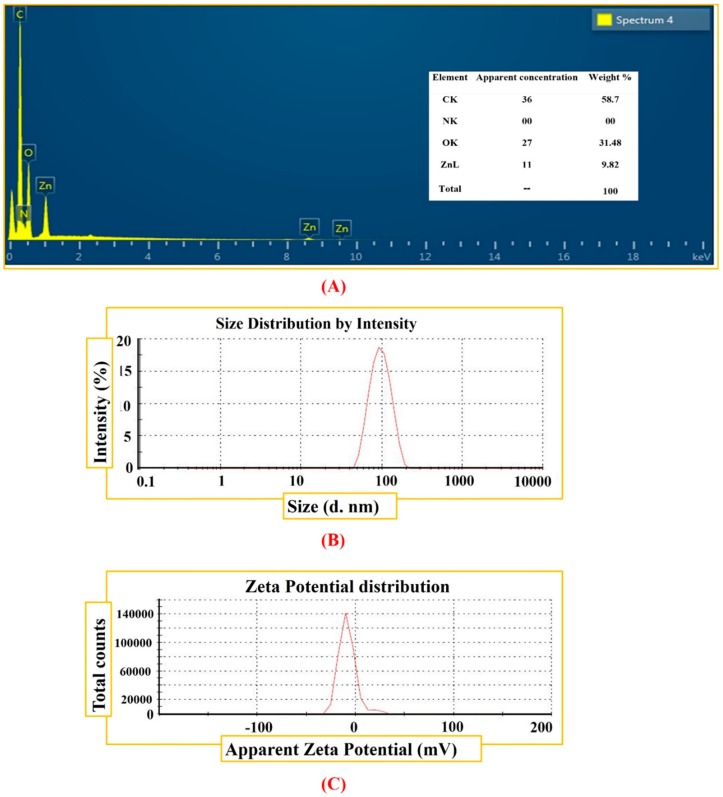
(**A**) Elemental composition using EDX (**B**) Size distribution of *Geranium wallichianum* mediated ZnONPs (**C**) Zeta potential measurement of ZnONPs.

The Raman and FTIR analyses were performed to determine the vibrational properties of ZnONPs. The distinct major modes of Raman spectra are located at 92.5 cm^−1^ (E_2_L), 180.77 cm^−1^ (2TAM), 313.23 cm^−1^ (2E2M), 403.04 cm^−1^ (E1TO), 565.12 cm^−1^ (E2H + E2L). Our results of Raman spectra of GW mediated ZnONPs are in agreement with previous studies using *S. thea* [44]. The Raman spectroscopy results are shown in Figure 3A. FTIR analysis was done to recognize the major functional group and their possible role in synthesis and stabilization of ZnONPs. The spectrum of leaves extract mediated zinc oxide nanoparticles is presented in Figure 3B. The peak centered at 3273.31 cm^−1^, 1395.87 cm^−1^, 1262.46 cm^−1^, 1060.47 cm^−1^ indicate O-H, C-C, -CH and C = C stretching. The other peaks located at 1565.48 cm^−1^ correspond to C−C. Peaks at 530 cm^−1^ signify Zn−O bond vibrations from ZnONPs. There was a shift in the peaks of biogenic ZnONPs which suggests that different functional groups of GW leaf extract are involved in synthesis of ZnONPs and prevent agglomeration [45]. The surface morphology of the biogenic ZnONPs was explored using SEM. The SEM images of biogenic ZnONPs are shown in Figure 4A–C. The smaller the size of nanoparticles, the larger the surface area and stronger the activity will be. Thus, ZnONPs have shown significant biological applications. Further, an insight into the morphology and size detail of ZnONPs was revealed by TEM. TEM image in (Figure 4D) shows that ZnONPs are hexagonal in shape with average size of ~18 nm which are consistent with calculation from XRD. Smaller size NPs with larger surface area have shown numerous applications especially in the field of medicine, chemo, and electrochemistry [46,47]. EDX spectra analysis revealed the surface chemical composition of biogenic ZnONPs. The EDX results have shown that all the ionic zinc was resulted into synthesis of ZnONPs leaving no ionic zinc peak. The EDX peaks showed that both Zn and oxygen exist in test samples while no other elements were observed in the EDX spectrum. The absence of other elements confirms the purity of biosynthesized ZnONPs. The EDX spectrum is shown in Figure 5A. The EDX pattern clearly shows that reduction of Zn salt with GW leaves extract yielding in crystalline ZnONPs. The size distributions, PDI and ζ-potentials (ZP) of thermally annealed ZnONPs were detected by DLS analyses. The results indicated larger particles aggregate of 98.26 nm. The ZP and PDI of ZnONPs were −8.53 mV and 0.232 (Figure 5B,C). The details about zeta size and ZP are given in Table 1. Our DLS results are similar to the previous report of ZnONPs using *Ixora coccinea* [48]. DLS is performed to confirm the size of NPs in colloid suspension in the range of nano and submicron, and its ZP measurement is based on particles movement under electric field. 

### 3.2. Bio-Potentials of Biogenic GW-ZnONPs

#### 3.2.1. Metabolic Activity of GW-ZnONPs against HepG2 Cells

**Figure 6:**
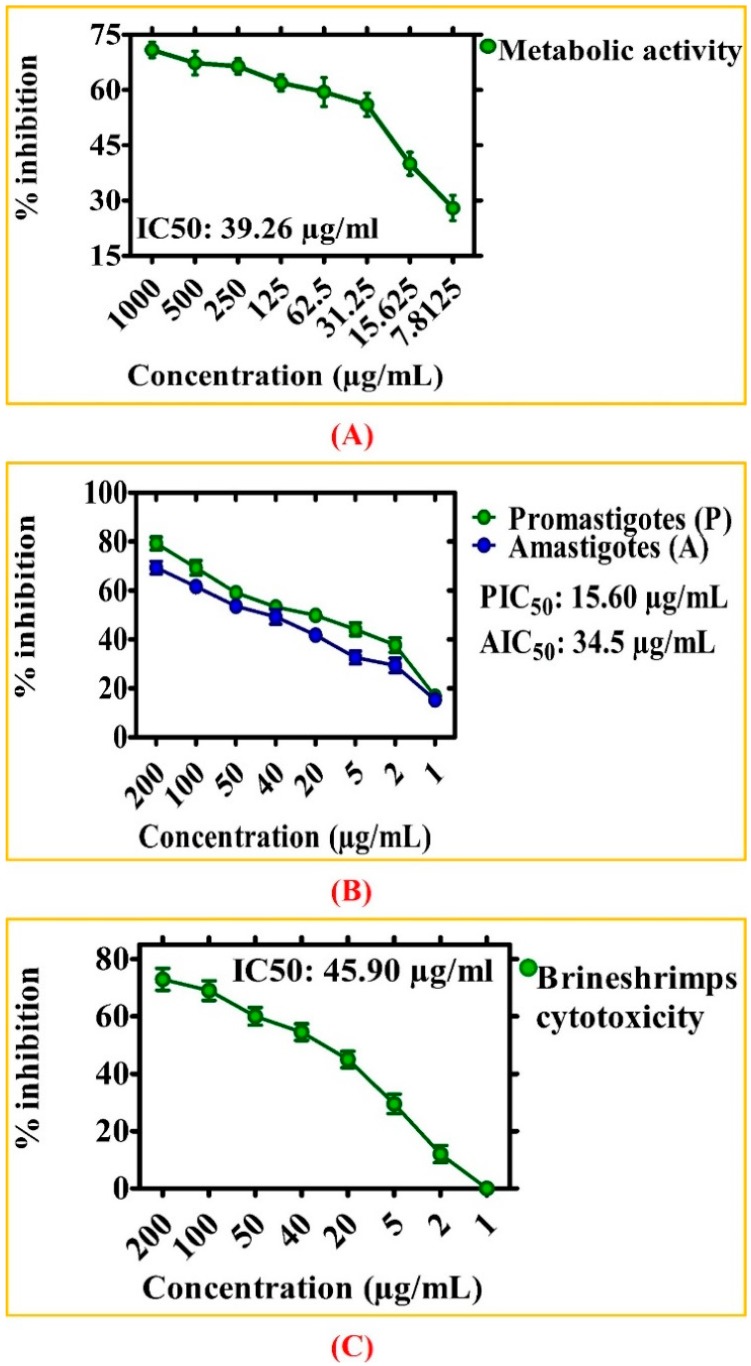
Cytotoxicity assays. The data in all figures represents the mean of three replicates (**A**) Cytotoxicity activities of *Geranium wallichianum* mediated ZnONPs against HepG2 cell line (**B**) Antileishmanial activities of ZnONPs (**C**) Cytotoxicity against brine shrimps.

Cancer is a fatal disease, a major cause of deaths around the globe, and is continuously increasing in cause of death by an estimated ~21 million by the year 2030 [30,49,50]. Among the numerous types of cancers, liver cancer is presently the second deadliest cancer in males and sixth in female causes (~745,517) deaths. The different risk factors related are viral infection, extensive alcohol use, and toxin exposures (aflatoxin) [51]. The cytotoxicity potential of the synthesized zinc oxide nanoparticles against liver cancer cells (HepG2) was evaluated using MTT cytotoxicity assay. The key results obtained by MTT cytotoxicity assay in HepG2 cells treated with various doses of ZnONPs ranging from 7.8125–1000 µg/mL for 48 h are summarized in Figure 6A. Our results of ZnONPs have determined strong reduction in the metabolic activity of HepG2 cancer cells. The metabolic activity was reducing continuously with increase in ZnONPs concentrations. The highest inhibition potential (~71% mortality) was achieved at 1000 µg/mL and cytotoxicity potency was decreasing with a decrease in concentration. The reduction in metabolic activity has shown that ZnONPs might have potential anticancer activity. The IC_50_ value recorded for GW mediated ZnONPs against HepG2 cell lines was 39.26 µg/mL. The cytotoxic effects induced by GW-NPs at lower concentrations could be due to the plant components attached to the ZnONPs. The results obtained from this study are also very well supported with various evidences for the cytotoxic effect of green ZnONPs using *Rhamnus virgata* leaf extract against the liver cancer HepG2 cell line in vitro [52,53].

#### 3.2.2. Antileishmanial Potential of ZnONPs

Leishmania is a tropical disease with an excessive epidemiological diversity caused by at least 20 *Leishmania* species and is transferred by the bite of female sandflies [54]. Leishmanial parasites have wide distribution range in ~100 countries around the world. The drugs present in commercial market for the treatment of leishmaniasis are generally toxic, less potent and expensive. As for example, Antimonials designed as a potential candidate for leishmaniasis therapy has lost its potential against Leishmanial parasites and has developed resistance against it. Thus, pharmaceutical industries are working hard in this direction to develop novel drugs which will be more effective, less toxic, and cost effective for the treatment. Significant research studies have been performed to design MNPs for the treatment of Leishmania. Various NPs have been used in diverse studies to determine cytotoxic potentials against *L*. parasites [34]. However, the biosynthesized ZnONPs are poorly studied for the treatment of *L. tropica*. 

In the present report, antileishmanial potentials of ZnONPs was evaluated using *L. tropica* (KMH23) Figure 6B. The *L. tropica* parasites were exposed to various doses (1–200 μg/mL) of ZnONPs for 72 h. The antileishmanial potentials of ZnONPs were increasing with an increase in concentrations of ZnONPs and determined significant antileishmanial potentials against *L. tropica* promastigotes with IC_50_: 15.60 µg/mL. Similarly, ZnONPs also shown significant potential against *L. tropica* amastigotes with IC_50_: 34.5 µg/mL which are consistent with earlier studies of biosynthesized ZnONPs [34,53]. The dose-dependent nature and low IC_50_ value signify that these ZnONPs can be utilized in drug delivery for the treatment of leishmaniasis.

#### 3.2.3. Antibacterial and Antifungal Activities

**Figure 7:**
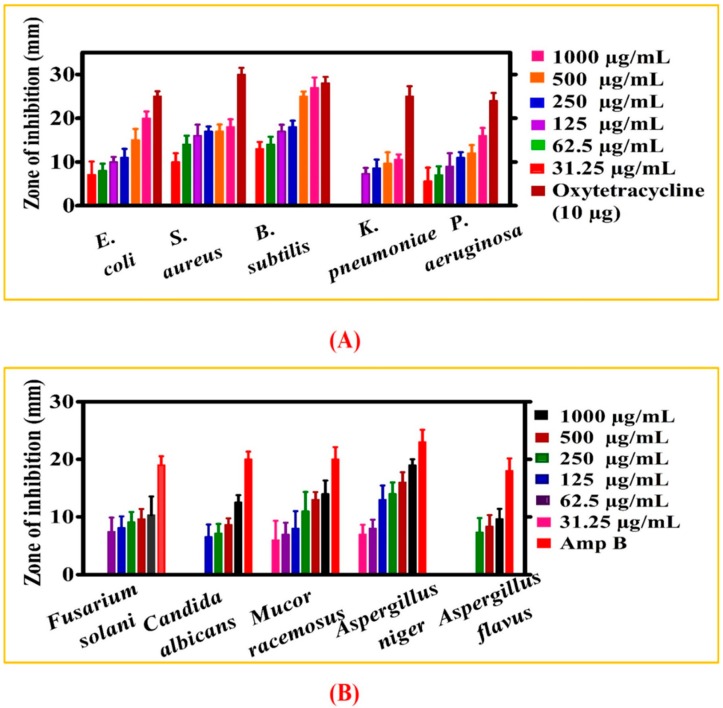
Antibacterial and antifungal assays. The data in all figures represents the mean of three replicates (**A**) Antibacterial potential of biogenic ZnONPs (**B**) Antifungal potential of biogenic ZnONPs.

The biogenic zinc oxide nanoparticles have also shown significant antibacterial potential against various bacterial strains (BS). For this purpose, different bacterial strains were treated with various doses of ZnONPs ranging from 31.25–1000 µg/mL Figure 7A. The activity was done against different gram positive (*S. aureus* and *B. subtilis*) and gram negative BS (*P. aeruginosa*, *K. pneumoniae*, *E. coli*). Most BS were found susceptible where ZnONPs have shown potential results by inhibiting them. *B. subtilis* was reported to be the most susceptible strain with MIC score of 7.8 µg/mL while *K. pneumoniae* was found to be the least susceptible strain with an MIC value of 125 µg/mL. Oxytetracycline was taken as positive control while treating BS to different doses of ZnONPs. No single dose has shown stronger potential than positive control. Overall, our ZnONPs have shown potential antibacterial activities against different BS which are in line with the previous reports of biosynthesized nanoparticles [28,55]. The increased antibacterial potential of ZnONPs is due to the bioactive functional groups attached on the surface of NPs. In a nutshell, ZnONPs concluded concentration dependent response against different strains of bacteria. Some other studies have discussed the antibacterial potentials of ZnONPs and shown that ROS generation is the core mechanisms that give antimicrobial potentials to NPs. Further, membrane damage (membrane protein damage) due to NPs absorption on surface result in bacterial cell damaging. Similarly, surface defect in the symmetry of NPs is responsible for bacteria inhibition and causes injury to cells [56]. Besides, different functional groups attached from GW leaves extract result in capped ZnONPs, which play an important role in the bacterial inhibition.

Furthermore, fungicidal potential of ZnONPs was evaluated using various fungal strains (FS). Before activity was performed different concentration of ZnONPs were prepared ranging from 31.25–1000 µg/mL. The drug amp-B was employed as positive control to compare inhibition potential of ZnONPs. Various FS were used; *Fusarium solani*, *M. racemosus*, *A. niger*, *A. flavus* and *C. albicans* (Figure 7B). Substantial work has been performed on antibacterial potentials of ZnONPs while limited fungicidal potentials have been reported for biogenic ZnONPs. Our current study for the first reported the fungicidal activity of GW-ZnONPs. For this purpose, different FS were treated with various concentrations of ZnONPs (31.25–1000 µg/mL) to determine their antifungal potentials. Generally, a dose dependent inhibition response was reported for ZnONPs where *A. flavus* was the least susceptible fungal strain (MIC: 250 µg/mL while *A. niger* and *M. racemosus* were the most susceptible strain with MIC: 31.25 µg/mL. Among the various FS, *M. racemosus*, *A. niger* and *F. solani* were inhibited at all doses. Besides, ROS, previous studies claim that interaction of ZnONPs with fungal hypea and spores leads to inhibition of their growth. Significant concentration mediated fungicidal assays are reported in previous studies using different fungal strains [34] and are consistent with our present GW-ZnONPs study. MIC values for various bacteria and fungus strain are presented in Table 2.

#### 3.2.4. Enzyme Inhibition Potentials of ZnONPs

**Figure 8:**
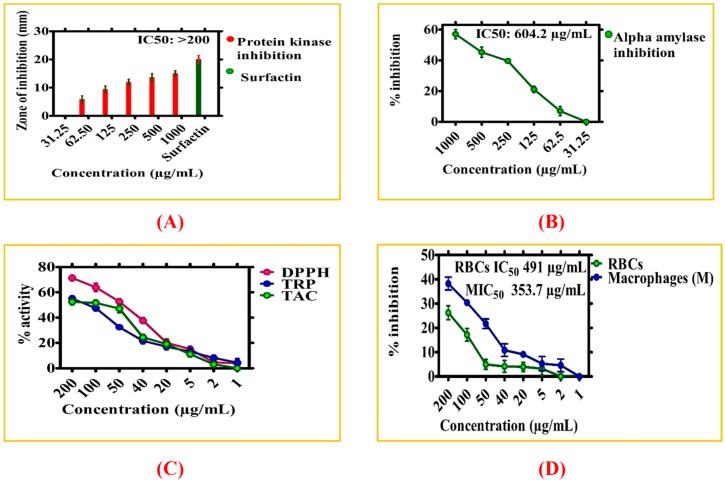
(**A**) Inhibition potential of *Geranium wallichianum* mediated ZnONPs against protein kinase (**B**) Inhibition potential against alpha amylase (**C**) Antioxidant potential of ZnONPs (**D**) Biocompatibility potential of ZnONPs against human RBCs and macrophages.

Figure 8A shows protein kinase (PK) enzyme inhibition potential of ZnONPs. These enzymes play significant role in phosphorylation of important amino acids; serine-threonine, tyrosine residues, regulate important processes inside cells like metabolism, apoptosis, proliferations, and differentiation. Deregulated phosphorylation of amino acids can result into genetic abnormalities and result in the development of cancer. Therefore, any substance with potentials to inhibit PK enzymes are of great attention in the field of cancer research [57]. PK phosphorylation has played an important role in the formation of hyphae in *Streptomyces* fungal strain and same mechanism is used to evaluate the PK inhibition potentials and is utilized to study medicinal compounds for determining PK inhibition [5]. The PK inhibition activity was done via disc diffusion method using different doses of ZnONPs ranging from 31.25−1000 µg/mL. The surfactin was taken as a positive control. Different ZOI were observed at different doses of ZnONPs, and the highest ZOI was measured as 15 mm at 1000 μg/mL, which shows important protein kinase inhibition potency of ZnONPs. A concentration-dependent activity is reported for ZnONPs. No single dose has shown stronger potential than the positive control. Therefore, a potential signal transduction inhibitor is identified in the form of nanoscaled ZnO that can be further exploited for anti-infective and anticancer properties. Because of the protein kinase inhibition property, one can pre-deduce that biosynthesized ZnONPs may play an important role in cancer therapeutics. Our results of PK inhibition are in line with the previous findings [53].

Besides PK inhibition assay, alpha amylase (AA) inhibition potency of ZnONPs was determined using various concentrations (31.25–1000 µg/mL) of ZnONPs. The AA play significant role by converting carbohydrates into glucose [57], therefore, blocking or by inhibiting the activity of alpha amylase can prevent the level of glucose formation; thus, it may provide new insights into a nano level treatment of diabetes [58]. In our study, biogenic ZnONPs were explored for their AA inhibition activity and have determined significant potential by the inhibition of AA. The highest inhibition rate was observed 57% at 1000 μg/mL, while AA inhibition potentials was slowly decreasing with a decrease in concentration of ZnONPs. Figure 8B shows the biological inhibition potential of ZnONPs against AA. The results of AA activity are in agreement with the previous findings [44,53].

#### 3.2.5. Antioxidant Activities of ZnONPs

Figure 8C indicates antioxidant potentials of ZnONPs. The antioxidant activities were evaluated in concentration ranging from 1–200 µg/mL. Maximum score for TAC of ZnONPs in terms of AA equivalent/milligrams was reported as 52.43% at 200 μg/mL. TAC assay is mainly used to evaluate the scavenging effect of tested chemicals towards reactive oxygen species (ROS). In the present study, water extract of GW leaves extract was utilized in the reduction and stabilizations of metal ions. It can be concluded from our antioxidant activities, that several phenolic compounds available in the GW leaves extract scavenge ROS which are attached on the surface of ZnONPs. 

To further explore the antioxidants species coated on the surface of biogenic nanoparticles, TRP assay was determined. This activity was done to study the reductones that play an important role in the antioxidant potential by providing H-atoms and causing damage to free radical chains [59]. The biosynthesized ZnONPs showed potential antioxidant activity. The reducing power of ZnONPs was decreasing with decrease in concentrations of ZnONPs. The maximum TRP (55%) was observed at its highest concentration of 200 μg/mL. Significant DPPH radicals scavenging activity (71.36%) was observed for ZnONPs at 200 μg/mL. From data presented in Figure 8C, it can be concluded that numerous antioxidants compound may be responsible in reduction and stabilization of ZnONPs via GW leaf extracts. Our antioxidants result of GW-mediated ZnONPs are consistent with the previous studies of ZnONPs via *S. thea* and *F. indica* [34,44]. The variations and disagreement compare to other studies may be due to various important factors like experiment condition, method of nanoparticles fabrication, plant, plant part used, and nanoparticle size, etc.

#### 3.2.6. Biocompatibility Potential Assays

The biocompatibility and toxicological effect of zinc oxide nanoparticles were determined using human macrophages and RBCs. Biological substance with hemolytic activity of greater than 5% are known as hemolytic, between 2–5% are slightly hemolytic, while less than 2% is non-hemolytic [60]. If a given nanoparticle is hemolytic, it will rupture red blood cells, which further result in hemoglobin release. To confirm the bio-safe nature, hemolysis assay was conducted using human RBCs. The RBCs were exposed to various concentrations of ZnONPs in a concentration ranging from 200–1 µg/mL. The data obtained have shown that the synthesized are non-hemolytic at lower concentration (2 μg/mL), slightly hemolytic at 5 to 50 μg/mL, while hemolytic at concentrations of >50 μg/mL. These results are in agreement to previous reports of *S. thea* mediated ZnONPs [44]. The IC_50_ value of ZnONPs against human red blood cells was recorded is 491 μg/mL. Our research study confirmed that biosynthesized ZnONPs are non-hemolytic and are considered biocompatible in low concentrations.

The biocompatibility assay was further confirmed by using human macrophages. For this purpose, human macrophages were seeded in 96-well plate and were cultured in RPMI media for 24 h for cells attachment. Further, cells were treated with various concentrations of ZnONPs (200–1 µg/mL). MTT cell viability assay was done to perform biocompatible nature of ZnONPs. The macrophage responded to ZnONPs treatment in a dose dependent manner. The results indicated that ZnONPs at 200 μg/mL inhibit growth of the macrophages by ~32% which determine the bio-safe behavior of biogenic ZnONPs. Normally, macrophages have established mechanisms to deal with ROS produce from external source. According to research studies, ROS are non-toxic to both RBCs and macrophages at a lower concentration unless concentration increases beyond that limit which will be considered toxic for RBCs and macrophages [61]. The IC_50_ value for ZnONPs was calculated >353.7 μg/mL. The results of biocompatibility assays of ZnONPs are presented in Figure 8D.

## 4. Conclusions and Future Perspectives

In summary, a simple, safe, ecofriendly, and one-step process was used for the biofabrication of ZnONPs utilizing GW *leaves* extract without utilizing any chemical reagents or surfactants. Different characterization techniques were performed. TEM analysis showed that ZnONPs was of ~18 nm with a hexagonal shape. Furthermore, different in vitro biological activities of ZnONPs were performed. The bio-potentials of ZnONPs were investigated against different pathogenic microbial strains and confirmed that ZnONPs have shown significant antimicrobial potential. The ZnONPs displayed strong anticancer and antileishmaniasis activities. Furthermore, moderate antioxidant and enzymes inhibition activities have been investigated. The biosafe nature of ZnONPs was confirmed using human RBCs and macrophages. Based on the above findings, we can say that green synthesis is the way forward and new frontier for designing nanomedicine and can be utilized in different theranostic applications for the treatment of different diseases. In addition, different in vivo studies are encouraged on toxicity aspects in different animal models, and once their biocompatibility and bio-safe nature is confirmed, only then can these NPs can be utilized in clinical applications. Further studies are encouraged on the mechanistic and synthesis aspects of the ZnONPs by using different medicinal plant materials.

## Figures and Tables

**Table 1:** Zeta potential measurements of the ZnONPs.

Zeta Size (d. nm) and Potential (mV)	
Zeta size	98.26 (d. nm)
Z-Average	98.09 (d. nm)
PdI	0.232
Intercept	0.943
Zeta potential	−8.53 mV
Zeta deviation	9.16 mV
Conductivity	0.00275 mS/cm
Result quality	Good

**Table 2:** MICs values of different bacterial and fungal strains.

Antibacterial Activity	
Bacterial Strain	MIC (µg/mL)
Gram Positive	
*B. subtilis* (ATCC: 6633)	7.8
*S. aureus* (ATCC: 25923)	15.625
Gram Negative	
*P. aeruginosa* (ATCC: 9721)	31.25
*E. coli* (ATCC:15224)	15.625
*K. pneumonia* (ATCC: 4617)	125
**Antifungal Activity**	
Fungal Strain	MIC (µg/mL)
*Aspergillus flavus* (FCBP: 0064)	250
*Aspergillus niger* (FCBP: 0918)	31.25
*Candida albicans* (FCBP: 478)	125
*Fusarium solani* (FCBP: 0291)	62.5
*Mucor racemosus* (FCBP: 0300)	31.25
